# Stromal mesenchyme cell genes of the human prostate and bladder

**DOI:** 10.1186/1471-2490-5-17

**Published:** 2005-12-12

**Authors:** Young Ah Goo, David R Goodlett, Laura E Pascal, Kelsey D Worthington, Robert L Vessella, Lawrence D True, Alvin Y Liu

**Affiliations:** 1 Department of Urology, University of Washington, Seattle, WA, USA; 2 Department of Medicinal Chemistry, University of Washington, Seattle, WA, USA; 3 Institute for Systems Biology, Seattle, WA, USA; 4 Puget Sound VA Health Care System, Seattle, WA, USA; 5 Department of Pathology, University of Washington, Seattle, WA, USA

## Abstract

**Background:**

Stromal mesenchyme cells play an important role in epithelial differentiation and likely in cancer as well. Induction of epithelial differentiation is organ-specific, and the genes responsible could be identified through a comparative genomic analysis of the stromal cells from two different organs. These genes might be aberrantly expressed in cancer since cancer could be viewed as due to a defect in stromal signaling. We propose to identify the prostate stromal genes by analysis of differentially expressed genes between prostate and bladder stromal cells, and to examine their expression in prostate cancer.

**Methods:**

Immunohistochemistry using antibodies to cluster designation (CD) cell surface antigens was first used to characterize the stromas of the prostate and bladder. Stromal cells were prepared from either prostate or bladder tissue for cell culture. RNA was isolated from the cultured cells and analyzed by DNA microarrays. Expression of candidate genes in normal prostate and prostate cancer was examined by RT-PCR.

**Results:**

The bladder stroma was phenotypically different from that of the prostate. Most notable was the presence of a layer of CD13^+ ^cells adjacent to the urothelium. This structural feature was also seen in the mouse bladder. The prostate stroma was uniformly CD13^-^. A number of differentially expressed genes between prostate and bladder stromal cells were identified. One prostate gene, proenkephalin (PENK), was of interest because it encodes a hormone. Secreted proteins such as hormones and bioactive peptides are known to mediate cell-cell signaling. Prostate stromal expression of PENK was verified by an antibody raised against a PENK peptide, by RT-PCR analysis of laser-capture microdissected stromal cells, and by database analysis. Gene expression analysis showed that PENK expression was down-regulated in prostate cancer.

**Conclusion:**

Our findings show that the histologically similar stromas of the prostate and bladder are phenotypically different, and express organ-specific genes. The importance of these genes in epithelial development is suggested by their abnormal expression in cancer. Among the candidates is the hormone PENK and the down-regulation of PENK expression in cancer suggests a possible association with cancer development.

## Background

The functional development of the prostate is governed by stromal mesenchyme induction and epithelial response. This stromal/epithelial interaction was demonstrated by heterotypic tissue recombinants engrafted in animal hosts in which the stromal element dictated the organogenesis of the implanted epithelial component [1]. For example, adult human bladder epithelial cells can be transdifferentiated into prostatic structures by prostate inductor [2]. In that study, neonatal rat seminal vesicle mesenchyme induced adult human urothelial cells to produce glandular structures resembling the prostate histologically and functionally, in which secretory-like cells in these structures produced prostate-specific antigen (PSA). The inductive mechanism is evolutionarily conserved because it was demonstrated with heterospecific mouse/human recombinants. Besides stromal/epithelial interaction, morphogenesis and functional cytodifferentiation are dependent on interactions between epithelium and basement membrane and the extracellular matrix [3]. Prostate development is also under hormonal control and the influence of androgen is primarily mediated by the stromal cells [4]. There is also evidence that stromal/epithelial interaction is involved in the differentiative development of other organs like the gut and kidney [5,6].

We postulate that organ-specific stromal cell genes are important factors in organ development. In order to identify these genes, we compared the expression profile of prostate stromal cells with that of bladder stromal cells. Since stromal cells of the two organs appear histologically indistinguishable we first used immunohistochemistry with a panel of CD antibodies to look for differences. Next, we used DNA array analysis to determine genes that are differentially expressed by the two stromal cell types. Due to experimental demands of RNA quantity we used stromal cells cultured in vitro. The organ specificity of the candidate genes was verified with RT-PCR analysis. Given the importance of stromal cells in epithelial differentiation, it is possible that diseases such as cancer of the epithelial cells could arise from defects in or a loss of stromal influence. We, therefore, also examined the expression of prostate stromal cell genes in cancer.

## Methods

### Prostate and bladder tissue, and stromal cell culture

Prostate tissue specimens were obtained from patients undergoing radical prostatectomy for their cancer treatment. Prostate metastases were obtained from patient donors after death through the Department of Urology tumor acquisition necropsy program [7]. Bladder tissue specimens were obtained from patients undergoing cystoprostatectomy for their bladder cancer. The cellular composition of these specimens was determined by histological examination of tissue block sections. For stromal mesenchyme (fibromuscular) cells, only specimens taken from cancer-free areas of the resected organs were used. Cultures were started either by placing tissue pieces on plates or by plating single cells prepared by tissue digestion with collagenase [8]. Briefly, fresh tissue specimens were minced and placed in RPMI-1640 media supplemented with 5% fetal bovine serum (FBS), 10^-8 ^M dihydrotestosterone (DHT), and digested with type I collagenase (Invitrogen, Carlsbad, CA, USA) overnight at room temperature. The digested tissue was filtered through a Falcon 70-μm filter (Becton-Dickinson, Franklin Lakes, NJ, USA) and aspirated with a 18-gauge needle. Stromal (STROM) and epithelial (EPI) cell types were partitioned by centrifugation in a discontinuous Percoll density gradient: STROM at ρ = 1.035 and EPI at ρ = 1.07. Prostate or bladder STROM cells from the Percoll gradients were cultured in RPMI-1640 supplemented with 10% FBS and 10^-8 ^M DHT. Cells were trypsinized and serially passaged, and harvested at the 4–5^th ^passage. Light microscopy was used to check the cell morphology. For confirmation, the cells were tested for expression of epithelial cytokeratins (none) and CD markers (CD90 positive) by immunohistochemistry and other cell type-specific markers by reverse transcriptase polymerase chain reaction (RT-PCR) as described in a previous report [8]. Percoll gradients were used additionally to remove contaminant epithelial cell types, if any, before RNA isolation for array analysis. The use of human material for research was approved by the Institutional Review Board of the University of Washington.

### Immunohistochemistry

Immunohistochemistry specifications, including experimental design, staining, and image analysis, were compliant with Minimum Information Specification For In Situ Hybridization and Immunohistochemistry Experiments (MISFISHIE) [9]. Serial 5-μm thin sections were prepared from frozen blocks of prostate and bladder tissue, and processed for immunostaining using a commercial kit (Vector Labs, Burlingame, CA, USA). Antibodies to CD13 (clone WM15) and other CD molecules (BD-PharMingen, San Diego, CA, USA) were used at a starting concentration of 8 ng/μl. The use of these antibodies has been described in previous reports [10,11]. A custom-made anti-PENK (proenkephalin) rabbit polyclonal antibody (Abgent, San Diego, CA, USA) was used at 3.5 ng/μl. The antibody was raised against a synthetic PENK peptide – C*TGDNRERSHHQDGSDNE – from amino acid residues T163 to E179, plus an extra C for conjugation to carrier [12]. The immunoreaction was carried out at room temperature for 30 min. Biotinylated anti-mouse IgG, anti-mouse IgM, or anti-rabbit IgG was used for chromogen detection. Immunostained sections were imaged with Olympus BX41 microscope (Melville, NY, USA) equipped with MircoFire digital camera (Optronics, Goleta, CA, USA). Composite images were constructed with Photoshop CS (Adobe Systems, San Jose, CA, USA) and all source images are available at our SCGAP website [13].

### Gene expression analysis by DNA microarray

A 40K human cDNA-gene chip (IMAGE Consortium, representing 35,013 UniGENE clusters) was used for gene expression analysis. Microarray fabrication, RNA preparation, labeling with Cy3/Cy5 dyes (Amersham Biosciences, Piscataway, NJ, USA), hybridization, and washing have been described previously [14]. Total cellular RNA was prepared and its quality and concentration were determined with Agilent 2100 Bioanalyzer (Agilent Technologies, Palo Alto, CA, USA). Approximately 50 μg RNA was used for labeling. The array experiment was performed in quadruplicate and a dye-flip was also included. Raw data was processed, log ratios were estimated, and the significance of change was determined by a maximum likelihood method [14]. Differential expression was statistically assessed by a λ value, and values > 35 were considered significant. Expression was confirmed by RT-PCR using gene-specific primer pairs and RNA prepared from a different batch of cultured prostate and bladder cells. Primer pair sequences for either prostate or bladder genes used are listed in Table 1.

**Table 1:** Primer pair sequences used in the study.

**Gene**	**5' primer**	**3' primer**
STC1	GACACTCAGGGAAAAGCATTCG	CTCATGGGATGTGCGTTTGAT
PENK	CAACTTCCTGGCTTGCGTAATG	AGGAACTTCTTTGGAGTAACTTTCGC
BMP2	CGGTCTCCTAAAGGTCGACCA	GTCACGGGGAATTTCGAGTTG
RAB27B	GGTTTATAATGCACAAGGACCGAA	CCACACACTGTTCCATTCGCT
MMP3	CTCACAGACCTGACTCGGTTCC	ATCGATTTTCCTCACGGTTGG
GALNT7	CTCAAGTCTGCTCTCAGCGAATATG	TTTTGGTAGATGTGTCCTACCCGA
TRO	GACACTCAGGGAAAAGCATTCG	CTCATGGGATGTGCGTTTGAT
RIS1	GTAAGCCCATTGAGTCCACGC	TCACTTGGTCGCCACCCCCGA
ChGn	TGCAGCAGTGCCTTTCGATAG	GTCGAAATAAGATGAGCCGTTTGA
TNC	CAGACATCACTGAAAATTCGGCTAC	GCAAAGATTCTCAGTGTGTATTCCG
EDNRB	GCAAACCGCAGAGATAATGACG	TCAAGATATTGGGACCGTTTCG
STC2	CAAGTCATTCATCAAAGACGCCTT	CCTTTCATTTCACCTCCGGATATC
BF	ACTCCATGGTCTTTGGCCCAG	AGTGGATTGCTCTGCACTCTG
GFRA1	ACAGCAGATTGTCAGATATATTCCGG	GCGAGATCTGCAGATGTAATTCG
IMPA2	TAGCATTGGATTTGCTGTTCGAC	TCCCGCCCATAGTTAATCGTCT
PTGIS	GGCTACCTGACTCTTTACGGAATTG	GGCTCTCACTCAGCACGCTATC
OSF2	CTGCTTATTGTTAACCCTATAAACGCC	TTGCTCTCCAAACCTCTACGGAT
EST	GAAGCAGAGCCATGACAATCG	CCATGACTTCCATGACAATCGTC
B2M	CACGTCATCCAGCAGAGAATGGAAAGTC	TGACCAAGATGTTGATGTTGGATAAGAG
αSMA	GCCTCTGGACGCACAACTGGCATCG	GTTTGCTGATCCACATCTGCTGGAAGG

### Laser-capture microdissection

Prostate stromal cells were isolated from tissue by laser-capture microdissection (LCM). Eight-μm thick sections of frozen tissue blocks were cut, immediately fixed in cold 95% ethanol, briefly stained with hematoxylin using HistoGene Staining (Arcturus Bioscience, Mountain View, CA, USA), and dehydrated in 100% ethanol followed by xylene, as described in the Arcturus HistoGene LCM Frozen Section Staining Kit. Around 5,000 stromal cells were captured using Arcturus PixCell II machine. Following microdissection, captured cells were lysed in Arcturus RNA Extraction Buffer. RNA was isolated using Arcturus PicoPure RNA Isolation Kit, and then treated with RNase-Free DNase (Qiagen, Valencia, CA, USA). The RNA was used to analyze by RT-PCR for PENK expression.

### Gene expression analysis of stromal genes in cancer

Gene expression analysis of matched normal/non-cancer and cancer of the prostate was analyzed by RT-PCR. Tumor specimens were excised from cancer foci in surgically resected glands, and non-cancer specimens were taken from cancer-free areas. The small pieces of tissue (~1 mm^3^) were placed in RNA*later *(Ambion, Austin, TX, USA) if not processed immediately, and were homogenized in a lysis buffer. RNA was precipitated by isopropanol for CP (prostate cancer or enriched for cancer) and NP (normal prostate) cDNA synthesis as described previously [15]. Tumor specimens from necropsies (usually performed within 3 h of death) were similarly processed. Because of tumor necrosis, specimens that produced poor quality RNA were not used. Primers for β2 microglobulin (B2M) and smooth muscle actin (αSMA) were used to monitor the suitability of the cDNA for analysis. Twenty NP and CP pairs were tested.

## Results

### CD phenotypes of prostate stroma *vs*. bladder stroma

**Figure 1:**
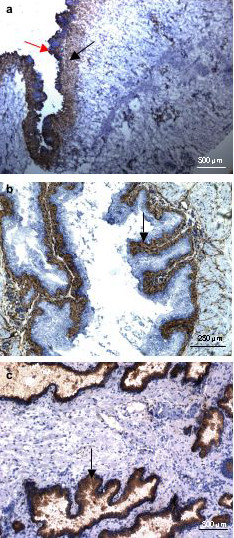
CD13 immunohistochemistry of the prostate and bladder. (**a**) Human bladder: CD13 stains a subpopulation of stromal cells (black arrow) in the lamina propria. The partially denuded urothelium is indicated by the red arrow. (b) Mouse bladder: CD13 also stains a similar region (black arrow) in the mouse bladder as in the human bladder. (c) Human prostate: CD13 stains only luminal epithelial cells (black arrow) of prostatic glands.

Almost all the constituent cell types of the prostate and bladder were identified by their particular complement of CD molecules. Overall, the stromal mesenchyme (fibromuscular) cells of the prostate and bladder were shown to share many of the CD molecules but there were differences as well. In particular, CD13 (aminopeptidase N) staining differentiated two sub-domains of the bladder lamina propria (Figure 1a). A region of not more than 10–20 cells in depth, by our estimate, next to the urothelium was positive for CD13 while the remainder of the lamina propria was negative. The CD13-positive part was termed superficial lamina propria by us. This CD13-positive superficial lamina propria was also found in the mouse bladder (Figure 1b). In contrast, the prostatic stromal cells showed no staining, and expression of CD13 was localized to the luminal epithelial cells as reported earlier (Figure 1c).

The reactivity of other CD antibodies is summarized in Table 2 for the bladder superficial lamina propria, bladder lamina propria, bladder muscularis, blood vessel smooth muscle cells and prostate stroma. Although smooth muscle cells are the principal cell types in these tissue structures they were distinguishable by their CD phenotypes. Besides CD13, differences between prostate and bladder stroma included CD51/61 (integrin αvβ3), CD56 (NCAM) – positive for prostate and negative for bladder, CD107a (LAMP-1) – negative for prostate and positive for bladder. In general, the bladder stromal mesenchyme compartment appeared to contain multiple cell types, such as CD13^+ ^*vs*. CD13^-^, CD49a^+ ^*vs*. CD49a^- ^(integrin α1), CD49d^+ ^*vs*. CD49d^- ^(integrin α4) compared to the uniformly CD13^-^/CD49a^+^/CD49d^- ^type in the prostate. All immunohistochemistry data are available at SCGAP website [13].

**Table 2:** CD phenotype of the prostate and bladder stromal fibromuscular cells.

	**bladder**				
			
	**superficial lamina propria**	**lamina propria**	**muscularis**	**blood vessel**	**prostate stroma**
CD13	+	-	+	+	-
CD29	+	+	+	+	+
CD30	-	-	+	-	+/-
CD40	-	-	+	-	-
CD47	+	+	-	+	+/-
CD49a	+ scattered	+ scattered	+/-	+	+ uniform
CD49d	-	+ scattered	-	-	-
CD49e	+ scattered	+ scattered	+	-	+
CD49f	-	-	-	+*	-
CD51/61	-	-	+	+	+
CD55	+	+	-	-	+
CD56	-	-	+	-	+
CD59	+	+ scattered	-	-	+
CD61	-	-	+	+	+
CD69	-	-	+/-	-	+/-
CD71	+	+	+	-	+
CD79a	+ scattered	+ scattered	+	-	+/-
CD81	+	+	?	-	+
CD90	+	+	-	+	+
CD97	+/-	-	+	-	-
CD99R	+	+	-	-	+
CD105	+	-	-	+	-
CD107a	+	+	-	-	-
CD112	-	+	-	-	-
CD131	+ scattered	+ scattered	+	?	+
CD151	+ scattered	+ scattered	+	-	+/-
CD184	+/-	+/-	+	+	+
CD243	-	-	+	-	-

* also CD109, CD31, CD34, CD93, CD141, LAP, LMP-1

+: stain

- : no stain

+/-: weak stain

? : equivocal (probably no stain)

### Organ-specific stromal mesenchyme cell genes

**Figure 2:**
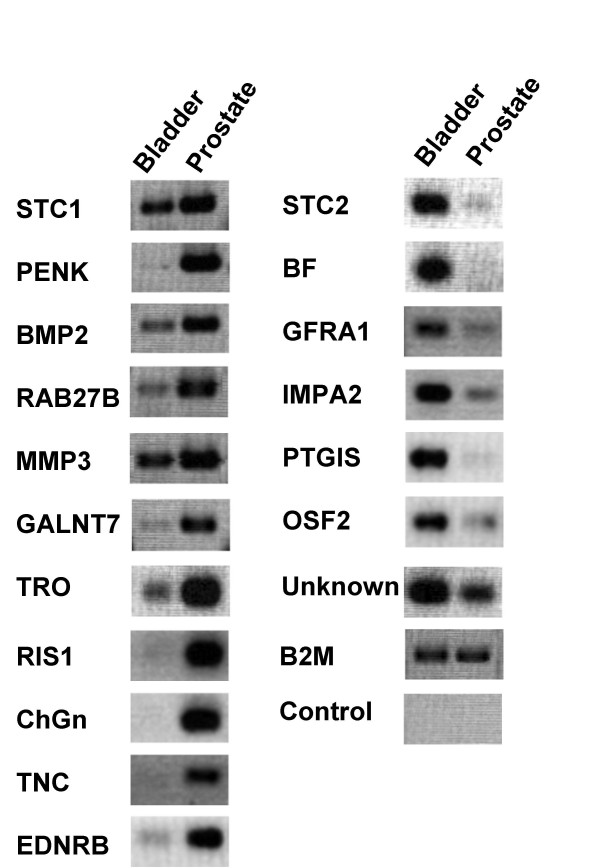
Organ-specific stromal genes. Shown are the results of the genes tested for their specificity, (cultured prostate *vs*. bladder stromal cells). Differential expression is gauged by the band intensity of the PCR products. B2M is β2-microglobulin, which was used as a positive control, and H_2_O was used as a negative control for the reaction.

The CD immunohistochemistry results showed that stromal cells of the prostate and bladder were different such that a transcriptome profiling would likely uncover more differentially expressed genes between them. Accordingly, stromal cells were cultured from fresh tissue taken from radical prostatectomy or cystoprostatectomy specimens. Because of their poor plating efficiency, epithelial cells (if present at the beginning) were outgrown by the stromal cells under the culture condition used. CD phenotyping of the resultant cultures showed previously that the prostate and bladder cells could be distinguished (e.g., reactivity to CD56) but a number of CD molecules, among them CD13, were also expressed as a result of cell culture [8]. Thus, it was not possible to state if the resultant bladder cells were derived from cells of the CD13^+ ^superficial lamina propria. RNA was obtained typically from ~10^6 ^cells harvested at the log phase. A 40K cDNA array was used to profile the transcriptomes of these cultured stromal cells. The list of the differentially expressed genes identified through this analysis is shown in Table 3, and differential expression of these genes between prostate and bladder was verified by RT-PCR (Figure 2). Among these was one hormone, proenkephalin (PENK), and this was studied further because hormones are likely important mediators of intercellular communication and proenkephalin is known to have a role in development. PENK was one of genes that showed the highest fold in differential expression between prostate and bladder (Table 3).

**Table 3:** Organ-specific stromal mesenchyme cell genes. Listed are the candidates identified through array analysis. Fold difference in the last column of each grouping provides a rough estimate of the level of differential expression between the two cell types.

**Prostate Stromal Genes**			**Bladder Stromal Genes**		
**Gene Description**	**Gene**	**Fold**	**Gene Description**	**Gene**	**Fold**

proenkephalin	PENK	26.41	B-factor, properdin	BF	44.23
stanniocalcin 1	STC1	20.86	argininosuccinate synthetase	ASS	19.36
trophinin	TRO	13.41	methylene tetrahydrofolate dehydrogenase (NAD^+ ^dependent), methenyltetrahydrofolate cyclohydrolase	MTHFD2	19.04
RAB27B, member RAS oncogene family	RAB27B	12.27	claudin 11 (oligodendrocyte transmembrane protein)	CLDN11	18.25
REV3-like, catalytic subunit of DNA polymerase z (yeast)	REV3L	10.68	interleukin 1, b	IL1B	17.57
CD59 antigen p18-20	CD59	10.19	carbonic anhydrase XII	CA12	16.49
matrix metalloproteinase 3 (stromelysin 1, progelatinase)	MMP3	10.06	pirin	PIR	15.52
homolog of rat orphan transporter v7-3	NTT73	9.12	osteoblast specific factor 2 (fasciclin I-like)	OSF-2	14.59
ras-induced senescence 1	RIS1	9.05	asparagine synthetase	ASNS	12.96
chondroitin b1,4 N-acetylgalactosaminyltransferase	ChGn	6.9	inositol(myo)-1(or 4)-monophosphatase 2	IMPA2	9.85
bone morphogenetic protein 2	BMP2	6.68	aldehyde dehydrogenase 1 family, member A1	ALDH1A1	9.48
sprouty homolog 4 (Drosophila)	SPRY4	6.29	prostaglandin I2 (prostacyclin) synthase	PTGIS	9.24
endothelin receptor type B	EDNRB	5.46	aldo-keto reductase family 1, member C1 (dihydrodiol dehydrogenase 1; 20-a (3-a)-hydroxysteroid dehydrogenase)	AKR1C1	9.2
interleukin 7 receptor	IL7R	5.44	BCL2 binding component 3	BBC3	9.06
tenascin C (hexabrachion)	TNC	5.29	chemokine (C-X-C motif) ligand 12 (stromal cell-derived factor 1)	CXCL12	8.65
tissue factor pathway inhibitor 2	TFPI2	5.13	activin A receptor, type II	ACVR2	8.03
plasminogen activator	PLAT	5.09	hyaluronan synthase 3	HAS3	7.34
transcription factor 21	TCF21	4.76	GDNF family receptor a1	GFRA1	7.12
KIAA1373 protein	KIAA1373	4.42	activating transcription factor 5	ATF5	6.31
UDP-N-acetyl-a-D-galactosamine:polypeptide N-acetylgalactosaminyltransferase 7	GALNT7	4.25	protein tyrosine phosphatase, receptor type, D	PTPRD	5.76
SEC14-like 2 protein (*S*. *cerevisiae*)	SEC14L2	3.54	growth arrest-specific 1	GAS1	5.49
gap junction protein, a7, 45kDa (connexin 45)	GJA7	3.21	Ca^2+^-dependent activator protein for secretion	CADPS	5.25
			paired-like homeodomain transcription factor 2	PITX2	4.86
			tryptophanyl-tRNA synthetase	WARS	4.76
			phosphogluconate dehydrogenase	PGD	4.56
			CUG triplet repeat, RNA binding protein 2	CUGBP2	4.46
			GATA binding protein 6	GATA6	4.4
			collectin sub-family member 12	COLEC12	3.94
			stanniocalcin 2	STC2	3.86

### Expression of PENK in prostate fibromuscular cells

**Figure 3:**
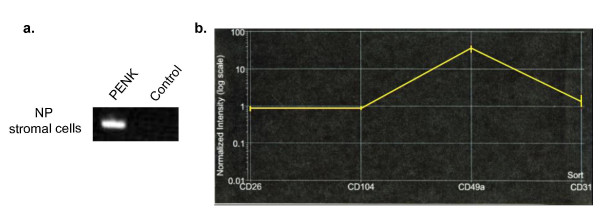
Expression of PENK in prostate stromal cell. (a) RT-PCR of laser-captured stromal cells. Cells were taken from non-cancer (NP), and PENK was detected in these cells. H_2_O was used as a negative control for the reaction. (b) PENK expression in sorted prostate cells. The various prostate cell types were sorted from tissue: CD26^+ ^luminal cells, CD104^+ ^basal cells, CD49a^+ ^stromal cells, and CD31^+ ^endothelial cells. Their transcriptomes were determined by microarray analysis using the Affymetrix Human Genome U133 Plus 2.0 GeneChips. PENK expression is localized to the CD49a^+ ^stromal cells.

The prostate stromal specific expression of PENK was verified by RT-PCR on stromal cells from tissue microdissected by laser-capture (Figure 3a). In our separate study, we had generated cell type-specific transcriptomes for prostate CD26^+ ^luminal cells, CD104^+ ^basal cells, CD49a^+ ^stromal cells, and CD31^+ ^endothelial cells through cell sorting and DNA array analysis (unpublished). Data analysis showed that PENK was expressed predominantly by the stromal cells (Figure 3b). These results showed that PENK expression was not due to cell culture.

**Figure 4:**
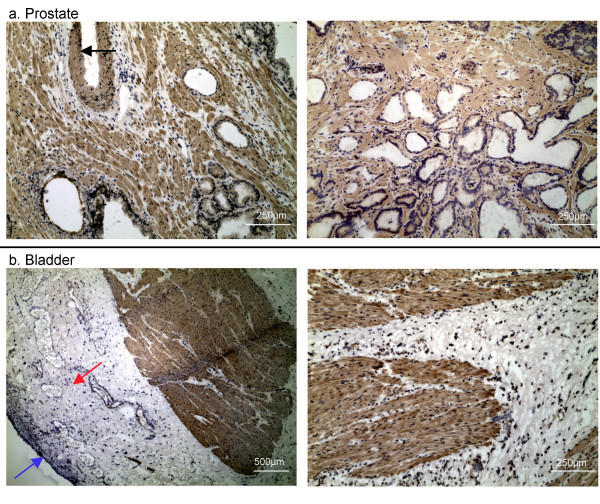
PENK immunohistochemistry of prostate and bladder. (**a**) In prostate, the PENK antibody stains the stroma in a pattern that is similar to that by CD56 [13]. The smooth muscle wall of a large blood vessel is also stained (black arrow, left panel). Benign glands appear to be stained at the luminal surface, but this staining is likely non-specific because it was present in the control without the primary antibody (in which the stromal staining was not seen). (**b**) In bladder, both the urothelium (blue arrow) and stroma (red arrow) of the lamina propria are not stained. Stained are the muscle bundles of the muscularis propria.

Proenkephalin is known to be processed into opioid pentapeptides, and these can be detected in the neural elements of the prostate by their specific antibodies [16]. The stromal PENK gene product was apparently not processed to the pentapeptide enkephalins as in neuronal cells. To localize PENK expression on tissue, a PENK peptide fragment was synthesized and used to immunize rabbits for anti-PENK antibodies. Immunohistochemistry of anti-PENK showed staining of stromal fibromuscular cells next to the prostate epithelium whereas no staining of the stromal cells adjacent to the urothelium was seen; although staining was evident in the muscularis (Figure 4). This result verified the differential expression of PENK between prostate and bladder.

### Down-regulation of PENK in prostate cancer

**Figure 5:**
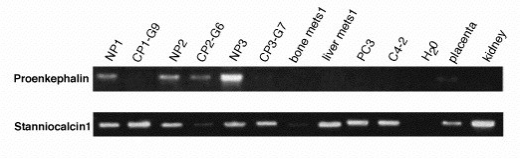
Expression of PENK and STC1 in prostate cancer. NP and CP are matched non-cancer and cancer specimens processed into cDNA. CP1 is a Gleason 4+5 (G9) tumor, CP2 a Gleason 3+3 (G6) tumor, and CP3 a Gleason 3+4 (G7) tumor. Bone and liver metastasis were obtained from end-stage diseases. PC3 and C4-2 are prostate cancer cell lines. PENK is detectable in all NP samples, it is lowered in G6, and barely detectable or absent in G7 and G9; as well as the metastases and cancer cell lines. PENK is found in placenta but not kidney. No significant differential expression was found for STC1 in these same samples, though the bone metastasis had lower expression than the liver metastasis, and lower expression in CP2 compared to NP2. STC1 is known to be expressed in the kidney. cDNA quantity of each sample used was monitored by B2M and αSMA (not shown).

Patient-matched CP and NP specimens were used for this analysis. The small size of the tissue was to ensure a purer sample of cancer without non-cancer tissue. In the result shown in Figure 5, the cancer specimens were primary tumors graded with a Gleason score of 3+3 (G6), 3+4 (G7), or 4+5 (G9). The bone and liver metastasis specimens were obtained from autopsies of donor patients. PENK was detected in all three NP samples, but its level was either decreased or undetectable in the CP and metastasis samples. Expression of smooth muscle actin was used as a control for the representation of stromal cells in NP and CP.

Stanniocalcin 1 (STC1) was another gene of interests identified through array analysis. It is also a hormone and known to have a role in urologic organ development. Like PENK, STC1 showed a large fold difference in expression between prostate and bladder (Table 3). The RT-PCR result of cultured cells showed that PENK was prostate-specific whereas STC1 was expressed more in prostate than bladder. The expression of STC1 was not significantly different between NP and CP. It is interesting to note that the prostate cancer cell lines, PC3 and C4-2, analyzed expressed STC1 (but not PENK), and it is thus possible that STC1 expression could be contributed by epithelial cells as well.

## Discussion

Our study showed that the stromal mesenchyme cells of the prostate and bladder are phenotypically and genotypically different, although they are indistinguishable by histomorphology. Within the bladder stroma of lamina propria are cells that are either CD13^+ ^or CD13^-^. The CD13^+ ^cells, in particular, constitute a discrete cell layer next to the urothelium. Because of their proximity to the urothelial cells these could be the cell type that functions in bladder stromal/epithelial interaction. For our differential gene expression analysis we relied on cultured cells to provide enough RNA for the array experiments. Since cell culture alters gene expression as shown by the CD profile of the cultured stromal cells [8], one question raised was if they could still be representative of the stromal cells in situ. For example, both cultured prostate and bladder stromal cells are CD13^+^. It is possible that the bladder cells were derived from the CD13^+ ^cells of the superficial lamina propria whereas the expression in prostate stromal cells was de novo. Despite this caveat, cultured cells were appropriate for our aim because cell-free conditioned media is known to contain the inductive factors for epithelial differentiation [17]. Furthermore, PENK expression in microdissected stromal cells confirmed that its expression is not due to in vitro culture. More recently, we have used cell sorting technology to determine the transcriptomes of prostate cell types, including the CD49a^+ ^stromal cells. The CD49a dataset allows us to further validate the stromal expression of PENK.

Prostate stromal production of PENK is notable because prostate luminal epithelial cells express CD10 (neutral endopeptidase) [10,11], and CD10 is known to have enkephalinase activity. Therefore, PENK and CD10 could constitute a signaling pathway in prostate stromal/epithelial interaction. PENK could potentially be one of the key factors that mediate prostate differentiation. PENK expression was reported in the literature to be in embryonic mesenchymal tissues during differentiation [18]. PENK is normally processed into Met-enkephalin and Leu-enkephalin, and antibodies to these pentapeptides could detect expression at nerve endings in the prostate [16]. The non-neural cell-derived stromal PENK is not likely processed to the known enkephalins. Our anti-PENK antibody showed that a PENK protein is indeed detectable in the stromal cells of the prostate, and not in those of the bladder. In the bladder, anti-PENK could detect a protein in cells of the muscularis (and cells of blood vessels).

We hypothesized that cancer could be due to defects in stromal/epithelial interaction, and expression of genes like PENK could be altered in cancer. This was shown to be the case by our RT-PCR analysis. In the tumor samples analyzed there was down-regulation of PENK expression. Previously, we showed that cancer epithelial cells are CD10 negative [11], which would further suggest a link between PENK and CD10 as defects in enkephalinase activity may be associated with decreased PENK expression in cancer. Decreased PENK expression may be due to gene methylation as reported in pancreatic cancer [19]. It is likely that stromal/epithelial interaction involves more than one gene, for example STC1. Stanniocalcins have a function in neuronal cell differentiation by regulating calcium and phosphate homeostasis [20]. STC1 is expressed during mouse urogenital development and has a role in the mesenchyme-epithelial interaction in organogenesis [21]. Both STC1 and STC2 are reported to be secreted as phosphoproteins from human fibrosarcoma, a tumor of mesenchymal cells [22]. Unlike PENK, STC1 did not show a clear altered pattern of expression between NP and CP. The cancer expression of several other prostate stromal genes was also investigated. GALNT7 and ChGn (Table 3), for example, also showed a similar expression pattern for NP and CP as PENK (unpublished). Further work will be carried out to confirm these initial results.

## Conclusion

The high throughput technology of genomics in conjunction with downstream analyses are critical to uncover the molecular basis of cell-cell interaction in normal development and cancer. In this study, we have identified candidate stromal cell genes for this process. Among these are genes that encode known hormones that function in intercellular signaling. The down-regulation of these genes in cancer suggests their importance in normal development.

## Competing interests

The author(s) declare that they have no competing interests.

## Authors' contributions

YG: study design, experiments, and drafting of the manuscript. DRG: study design. LEP: immunohistochemistry study. KDW: RT-PCR analysis. RLV: tissue acquisition and cDNA preparation. LDT: immunohistochemistry and pathology. AYL: study conception, study design and coordination. All authors have read and approved the final manuscript.

## Pre-publication history

The pre-publication history for this paper can be accessed here:


